# Supplementary material for: Content-rich biological network constructed by mining PubMed abstracts
Source: BMC Bioinformatics. 2004 Oct 8;5:147. doi: 10.1186/1471-2105-5-147 (PMC528731; doi:10.1186/1471-2105-5-147)
Supplement: Additional File 2 — The original results of the above study (non-essential files are deleted to keep the file size under the limit set by BMC bioinformatics). [file 1471-2105-5-147-S2.bz2 › chilibotAdditionalFile2/dip50/748ID9811664E18130/html/EXBD_TONB.html]

 


 **EXBD** and **TONB** 
  
Found 54 abstracts in PubMed, retrieved 50.  
 

 What does Google say? 
 PDF only 
| .edu only 

---

**Interactive relationship** (e.g. stimulation, inhibition, etc)

**Neutral relationship**- The cytoplasmic membrane proteins ExbB and  **ExbD**  support  **TonB**  dependent active transport of iron siderophores and vitamin B12 across the essentially unenergized outer membrane of Escherichia coli.  Ref: 9811664 J Bacteriol, 1998
- Interactions in the  **TonB**  dependent energy transduction complex ExbB and  **ExbD**  form homomultimers.  Ref: 9811664 J Bacteriol, 1998
- Energy for active transport is obtained from the proton motive force of the inner membrane through physical interaction with  **TonB**  ExbB  **ExbD** , an inner membrane complex.  Ref: 9886293 Nat Struct Biol, 1999
- The aim of this review is to describe an in vivo assay of the interactions taking place in the Tol Pal or  **TonB**  ExbB  **ExbD**  envelope complexes in the periplasm of Escherichia coli and between them and colicins or g3p protein of filamentous bacteriophages.  Ref: 12423784 Biochimie< MedlineTA>Biochimie, 1999
- ExbB and  **ExbD**  proteins are part of the  **TonB**  dependent energy transduction system and are encoded by the exb operon in Escherichia coli.  Ref: 12193634 J Bacteriol, 2002
- Both ExbB and  **ExbD**  were also required for association of  **TonB**  with the cytoplasmic membrane.  Ref: 12193634 J Bacteriol, 2002
- These results were consistent with the idea that ExbB and  **ExbD**  play roles in the assembly of  **TonB**  into an energy transducing complex.  Ref: 7642501 J Bacteriol, 1995
- The  **tonB**  Bb gene was situated upstream of exbB and  **exbD**  homologues and downstream of a putative Fur regulated promoter.  Ref: 10517598 Microbiology, 1999
- Specifically, both ExbB and  **ExbD**  were found to be required for  **TonB**  to conformationally respond to proton motive force.  Ref: 12193634 J Bacteriol, 2002
- A second  **tonB**  gene in Pseudomonas aeruginosa is linked to the exbB and  **exbD**  genes.  Ref: 10689178 FEMS Microbiol Lett, 2000
- While interaction between  **TonB**  and  **ExbD**  was suggested by the effect of  **ExbD**  on  **TonB**  stability, interaction of  **ExbD**  with  **TonB**  was detected by neither in vivo cross linking assays nor genetic tests for competition.  Ref: 7642501 J Bacteriol, 1995
- Cloning the mutation in the PhoA5 strain and sequencing the region adjacent showed that the mini Tn5 transposor interrupted the  **exbD**  gene, which forms part of the exbBD  **tonB**  operon.  Ref: 11514511 J Bacteriol, 2001
- It is proposed that the  **TonB**  ExbB  **ExbD**  complex opens via an energized conformation of the  **TonB**  protein channels in the outer membrane, formed by proteins which serves as highly specific binding sites for the various ferric siderophores and vitamin B12.  Ref: 7654405 FEMS Microbiol Rev, 1995
- This genetic organization has been reported in Haemophilus influenzae, H. ducreyi, Pseudomonas putida and Vibrio cholerae, and may represent a separate lineage of evolution from that of the Enterobacteriaceae in which  **tonB**  is unlinked with the accessory genes exbB and  **exbD** .  Ref: 9074497 Gene, 1997
- These data further support the concept that  **TonB** , ExbB, and  **ExbD**  form a complex in which the energized conformation of  **TonB**  opens the channels in the outer membrane receptor proteins.  Ref: 8631671 J Bacteriol, 1996
- both were eluted with the ExbB labeled with 6 histidine residues, demonstrating interaction of ExbB with  **ExbD**  and  **TonB** .  Ref: 8631671 J Bacteriol, 1996
- The N. meningitidis  **TonB**  protein is 28.8 to 33.6% identical to other gram negative  **TonB**  proteins, while the N. meningitidis  **ExbD**  protein shares between 23.3 and 34.3% identical amino acids with other  **ExbD**  and TolR proteins.  Ref: 9006036 J Bacteriol, 1997
- In this paper we show that  **ExbD**  protein, encoded by an adjacent gene in the exb cluster at 65, was also required for  **TonB**  dependent energy transduction and, like ExbB, was required for the stability of  **TonB** .  Ref: 7642501 J Bacteriol, 1995

**Non-interactive relationship** (e.g. studied together, co-existance, homology, etc.)

- It belongs to the group B colicins the uptake of which through the outer membrane depends on the  **TonB** , ExbB and  **ExbD**  proteins.  Ref: 8341256 Mol Gen Genet, 1993
- Evidence that the absence of ExbB or  **ExbD**  differentially affected the half lives of newly synthesized and steady state  **TonB**  was obtained.  Ref: 7642501 J Bacteriol, 1995
- In this study, we report the per cell numbers of  **TonB** , ExbB,  **ExbD**  and FepA for cells grown under iron replete and iron limited conditions.  Ref: 11967085 Mol Microbiol, 2002
- Cloning and functional characterization of Neisseria gonorrhoeae  **tonB** , exbB and  **exbD**  genes.  Ref: 9140974 Mol Microbiol, 1997
- Transcription initiation of the fec transport genes required the Ton system, consisting of  **TonB** , ExbB, and  **ExbD** , and was inhibited by carbonylcyanide m chlorophenylhydrazone CCCP and dinitrophenol DNP, which dissipate the electrochemical potential of the cytoplasmic membrane.  Ref: 9044267 Mol Microbiol, 1997
- Putative structural genes of the beta subunit of the histone like protein HU and of a new two component regulatory system were identified upstream from  **tonB**  and downstream from  **exbD** , respectively.  Ref: 10722583 Infect Immun, 2000
- Thus, the sequence comparison identified the ORF1, ORF2 and ORF3 as gonococcal homologues of the E. coli  **tonB** , exbB and  **exbD**  genes.  Ref: 9140974 Mol Microbiol, 1997
- A comparison of the DNA flanking the exbB  **exbD**   **tonB**  locus in P. haemolytica A1 and H. influenzae showed that the flanking regions are completely different between the two organisms.  Ref: 9074497 Gene, 1997
- Rather, FecA and the  **TonB** , ExbB and  **ExbD**  proteins are involved in induction of the fec transport genes independent of their role in ferric citrate transport across the outer membrane.  Ref: 7729419 EMBO J, 1995
- Cross linking analysis of epitope tagged ExbB and  **ExbD**  proteins with  **TonB**  antisera afforded detection of a previously unrecognized  **TonB**   **ExbD**  cross linked complex and confirmed the composition of the  **TonB**  ExbB cross linked complex.  Ref: 9811664 J Bacteriol, 1998
- bound to a nickel nitrilotriacetic acid agarose column retained  **ExbD**  and  **TonB**  specifically.  Ref: 8631671 J Bacteriol, 1996
- Based on sequence similarities, the Bordetella pertussis  **tonB**  exbB  **exbD**  locus was identified on a cloned DNA fragment.  Ref: 10722583 Infect Immun, 2000
- In gram negative bacteria, high affinity iron uptake requires the  **TonB**  ExbB  **ExbD**  envelope complex to release iron chelates from their specific outer membrane receptors into the periplasm.  Ref: 10722583 Infect Immun, 2000
- Analysis of sequence alignments of TolQ and TolR homologues from numerous Gram negative bacterial genomes, together with analyses of the different tolQ tolR mutants, revealed that the TM domains of TolQ and TolR present structural and functional homologies not only to ExbB and  **ExbD**  of the  **TonB**  system but also with MotA and MotB of the flagellar motor.  Ref: 11722743 Mol Microbiol, 2001
- In gram negative organisms, high affinity transport of iron substrates requires energy transduction to specific outer membrane receptors by the  **TonB**  ExbB  **ExbD**  complex.  Ref: 12562789 J Bacteriol, 2003
- Group B colicins, ferric siderophores and vitamin B12 are taken up via the  **TonB**  ExbB  **ExbD** , group A colicins via the TolA TolQ TolR system.  Ref: 8316079 Mol Microbiol, 1993
- Pasteurella multocida exbB,  **exbD**  and  **tonB**  genes are physically linked but independently transcribed.  Ref: 12044675 FEMS Microbiol Lett, 2002
- Energy coupled transport across the outer membrane of Escherichia coli ExbB binds  **ExbD**  and  **TonB**  in vitro, and leucine 132 in the periplasmic region and aspartate 25 in the transmembrane region are important for  **ExbD**  activity.  Ref: 8631671 J Bacteriol, 1996
- Neisseria meningitidis  **tonB** , exbB, and  **exbD**  genes Ton dependent utilization of protein bound iron in Neisseriae.  Ref: 9006036 J Bacteriol, 1997
- Only infection by phage T5 occurs independently of  **TonB** , ExbB and  **ExbD** .  Ref: 8039674 FEMS Microbiol Lett, 1994
- Reverse transcription RT PCR, using RNA from organisms grown under iron replete and iron restricted conditions, revealed that  **tonB** , exbB,  **exbD** , tbpB and tbpA are transcribed as a single unit with expression being up regulated in response to iron restriction.  Ref: 12742718 Vet Microbiol, 2003
- Active transport of Fe3 as ferrichrome complex through the outer membrane of Escherichia coli is mediated by the FhuA outer membrane protein and the  **TonB**  ExbB  **ExbD**  protein complex in the cytoplasmic membrane.  Ref: 7515827 FEBS Lett, 1994
- group B colicins use the proteins  **TonB** , ExbB, and  **ExbD** .  Ref: 7545150 J Bacteriol, 1995
- The Ton system consisting of the proteins  **TonB** , ExbB and  **ExbD**  is required for colicin and microcin import.  Ref: 12423780 Biochimie< MedlineTA>Biochimie, 1995
- Evolutionary relationship of uptake systems for biopolymers in Escherichia coli cross complementation between the  **TonB**  ExbB  **ExbD**  and the TolA TolQ TolR proteins.  Ref: 8316079 Mol Microbiol, 1993
- Transport of heme by the ShuA protein requires  **TonB**  and its accessory proteins ExbB and  **ExbD** .  Ref: 9393841 Infect Immun, 1997
- Vibrio cholerae iron transport haem transport genes are linked to one of two sets of  **tonB** , exbB,  **exbD**  genes.  Ref: 9781885 Mol Microbiol, 1998
- It is postulated that FecA,  **TonB** , ExbB and  **ExbD**  transfer the signal across the outer membrane, while the regulatory protein FecR transmits the signal across the cytoplasmic membrane to FecI in the cytoplasm.  Ref: 7729419 EMBO J, 1995
- Since no energy source is known in the outer membrane it is assumed that energy is provided through the action of the  **TonB** , ExbB and  **ExbD**  proteins, which are anchored to the cytoplasmic membrane.  Ref: 7688295 EMBO J, 1993
- In addition, outer membrane receptors together with the  **TonB**  ExbB  **ExbD**  complex are directly involved in induction of the transcription of ferric citrate and pseudobactin transport genes of Escherichia coli and Pseudomonas putida, respectively.  Ref: 7654405 FEMS Microbiol Rev, 1995
- Efficient usage of heme by N. meningitidis  **tonB**  and  **exbD**  mutants suggests the existence of a Ton independent heme utilization mechanism.  Ref: 9006036 J Bacteriol, 1997
- , Triton X 100 TX 100 did not require the  **TonB** , ExbB, or  **ExbD**  protein.  Ref: 11796379 Antimicrob Agents Chemother, 2002
- The energy of the proton motive force is postulated to be transduced to the transport proteins by a protein complex that consists of the  **TonB** , ExbB, and  **ExbD**  proteins.  Ref: 11566987 J Bacteriol, 2001
- The electrochemical potential of the cytoplasmic membrane CM energizes transport across the outer membrane, which requires an energy transducing device, consisting of the proteins  **TonB** , ExbB and  **ExbD** , from the CM to the OM.  Ref: 9377472 Biol Chem, 1997
- Other biochemical evidence also shows that the proteins that provide energy for iron transport at the outer membrane, such as the  **TonB**  ExbB  **ExbD**  system, are structurally very similar across bacterial species.  Ref: 11895294 Curr Top Med Chem, 2001
- In the absence of ExbB or  **ExbD** , newly synthesized  **TonB**  was degraded with a half life of 5 to 10 min, while the half life of  **TonB**  under steady state conditions was significantly longer, approximately 30 min.  Ref: 7642501 J Bacteriol, 1995
- Quantification of known components of the Escherichia coli  **TonB**  energy transduction system  **TonB** , ExbB,  **ExbD**  and FepA.  Ref: 11967085 Mol Microbiol, 2002
- The activity of the FhuA receptor in the outer membrane of Escherichia coli is dependent on the  **TonB** , ExbB and  **ExbD**  proteins which are anchored to the cytoplasmic membrane.  Ref: 8039674 FEMS Microbiol Lett, 1994
- Analysis of the nucleotide sequence in the complementing DNA showed that it codes for three genes in the order of exbB  **exbD**   **tonB** .  Ref: 9074497 Gene, 1997
- Excision of segments as small as 12 amino acids in the largest surface loop of FhuA converted FhuA into an open channel through which ferrichrome and antibiotics diffused independent of  **TonB**  ExbB  **ExbD** .  Ref: 7515827 FEBS Lett, 1994
- Cloning and characterization of the exbB  **exbD**   **tonB**  locus of Pasteurella haemolytica A1.  Ref: 9074497 Gene, 1997
- Characterization of the exbBD operon of Escherichia coli and the role of ExbB and  **ExbD**  in  **TonB**  function and stability.  Ref: 7642501 J Bacteriol, 1995
- Two accessory proteins, ExbB and  **ExbD** , are required for  **TonB**  function and it has been suggested that  **TonB**  and ExbBD form a complex in the membrane.  Ref: 8100348 Mol Microbiol, 1993
- Transport of bound chelates across the outer membrane depends upon  **TonB**  ExbB  **ExbD** , a cytoplasmic membrane localized complex that transduces energy from the proton motive force to high affinity receptors in the outer membrane.  Ref: 9643536 Mol Microbiol, 1998
- Escherichia coli and related Gram negative bacteria contain an energy coupled transport system through the outer membrane which consists of the proteins  **TonB** , ExbB,  **ExbD**  anchored in the cytoplasmic membrane and receptors in the outer membrane.  Ref: 8316089 Mol Microbiol, 1993
- The reconstitution in Escherichia coli of the S. marcescens Has system demonstrated that haem uptake is dependent on the E. coli ExbB,  **ExbD**  and  **TonB**  proteins.  Ref: 11737642 Mol Microbiol, 2001
- Following incubation on media containing hemoglobin, N. meningitidis IR3436  **tonB**  exbB  **exbD**  deletion mutant and N. gonorrhoeae PD3401  **tonB**  insertional mutant give rise to colonies which can grow with hemoglobin.  Ref: 10986265 J Bacteriol, 2000
- A complex of three membrane spanning proteins  **TonB** , ExbB and  **ExbD**  couples the chemiosmotic potential of the cytoplasmic membrane with siderophore uptake across the outer membrane.  Ref: 12409204 Biochim Biophys Acta, 2002
- A putative Fur binding sequence located upstream from  **tonB**  was detected in a Fur titration assay, indicating that the  **tonB**  exbB  **exbD**  operon may be Fur repressed in high iron growth conditions.  Ref: 10722583 Infect Immun, 2000
- Energy from the cytoplasmic membrane is transferred to the outer membrane with the aid of the Ton system, consisting of the proteins  **TonB** , ExbB, and  **ExbD** .  Ref: 8631671 J Bacteriol, 1996
- Activity of ExbB  **ExbD**  via TolA was higher in cells lacking  **TonB** , and activity of TolQ TolR via  **TonB**  was increased when TolA was missing.  Ref: 8316079 Mol Microbiol, 1993
- Sequencing of the Ton system genes revealed that the arrangement of the genes was exbB  **exbD**   **tonB** .  Ref: 9423852 Infect Immun, 1998
- When tested on soybean host plants, hmuT hmuR and  **exbD**   **tonB**  mutants exhibited wild type symbiotic properties.  Ref: 11532144 Mol Microbiol, 2001
- The exbB,  **exbD**  and  **tonB**  genes of the Pasteurella multocida animal pathogen have been cloned by complementation of an Escherichia coli  **tonB**  mutant.  Ref: 12044675 FEMS Microbiol Lett, 2002
- Crystal structures of two outer membrane transport proteins, FhuA and FepA, and biochemical data reveal strong long range conformational changes of the proteins upon binding of Fe 3 siderophore complexes and in response to energy transfer from the cytoplasmic membrane into the outer membrane via the  **TonB**  ExbB  **ExbD**  protein complex.  Ref: 11934617 Curr Opin Microbiol, 2002
- **TonB**  CysExbB  **ExbD**  D25N was still able to cross link with the FecA DALTV Cys derivatives in a  **tonB**  tolQ background, even though ExbD25N renders the  **TonB**  ExbBD complex nonfunctional V.  Ref: 12618451 J Bacteriol, 2003
- However, inducible levels of TX 100 resistance in gonococci had an absolute requirement for the  **TonB**  ExbB  **ExbD**  system, suggesting that such resistance in gonococci has an energy requirement above and beyond that required for constitutive pump activity.  Ref: 11796379 Antimicrob Agents Chemother, 2002
- **TonB** , the energy transducer, appears to go through a cycle during energy transduction, with the absence of both ExbB and  **ExbD**  creating blocks at two points i in the inability of  **TonB**  to respond to the cytoplasmic membrane proton motive force and ii in the conversion of  **TonB**  from a high affinity outer membrane association to a high affinity cytoplasmic membrane association.  Ref: 12193634 J Bacteriol, 2002
- PCR approaches allowed the amplification, cloning and sequencing of A. suis  **tonB** , exbB,  **exbD** , tbpB and tbpA homologues.  Ref: 12742718 Vet Microbiol, 2003
- In Gram negative bacteria, the  **TonB**  ExbB  **ExbD**  inner membrane multiprotein complex is required for active transport of diverse molecules through the outer membrane.  Ref: 11737642 Mol Microbiol, 2001
- The ratio of these proteins was calculated at one  **TonB**  two  **ExbD**  seven ExbB under all four growth conditions tested.  Ref: 11967085 Mol Microbiol, 2002
- ExbB and  **ExbD**  each formed two unique cross linked complexes which were not dependent on the presence of  **TonB** , the outer membrane receptor protein FepA, or the other Exb protein.  Ref: 9811664 J Bacteriol, 1998
- Allelic replacement of the wild type H. ducreyi exbB,  **exbD** , and  **tonB**  loci with the exbB,  **exbD** , and  **tonB**  deletion resulted in an H. ducreyi isogenic mutant unable to utilize hemoglobin but able to utilize hemin at the same levels as the parent strain to fulfill its heme requirement.  Ref: 9423852 Infect Immun, 1998
- Energy flows from the cytoplasmic into the outer membrane via a complex consisting of the  **TonB** , ExbB and  **ExbD**  proteins which are anchored in the cytoplasmic membrane.  Ref: 7654405 FEMS Microbiol Rev, 1995
- Nucleotide sequence analysis of DNA fragments subcloned from the Hb complementing cosmids identified four open reading frames, three of them homologous to Pseudomonas putida, E. coli, and Haemophilus influenzae exbB,  **exbD** , and  **tonB**  genes.  Ref: 9006036 J Bacteriol, 1997
- They share homology with the ExbB,  **ExbD** , and  **TonB**  proteins, respectively.  Ref: 11418549 J Bacteriol, 2001
- The energy is transduced to FecA by the inner membrane complex,  **TonB** , ExbB, and  **ExbD** .  Ref: 12618451 J Bacteriol, 2003
- The signal elicited by ferric citrate crosses the outer membrane via  **TonB** , ExbB, and  **ExbD** .  Ref: 8602163 Mol Gen Genet, 1996
- It is thought that the proteins  **TonB** , ExbB, and  **ExbD**  function as a coupling device between the cytoplasmic membrane and the outer membrane.  Ref: 9457864 J Bacteriol, 1998
- In contrast, E. coli  **tonB**  G174R, V178I was sensitive to colicin Ia and resistant to colicin B unless  **TonB** , ExbB and  **ExbD**  were overproduced which resulted in colicin B sensitivity.  Ref: 8039672 FEMS Microbiol Lett, 1994
- ExbB and  **ExbD**  do not function independently in  **TonB**  dependent energy transduction.  Ref: 12193634 J Bacteriol, 2002
- Inducible, but not constitutive, resistance of gonococci to hydrophobic agents due to the MtrC MtrD MtrE efflux pump requires  **TonB**  ExbB  **ExbD**  proteins.  Ref: 11796379 Antimicrob Agents Chemother, 2002
- Vibrio cholerae was found to have two sets of genes encoding  **TonB** , ExbB and  **ExbD**  proteins.  Ref: 9781885 Mol Microbiol, 1998
- In this study, Escherichia coli  **TonB**  was found to be distributed in sucrose density gradients approximately equally between the cytoplasmic membrane and the outer membrane fractions, while two proteins with which it is known to interact, ExbB and  **ExbD** , as well as the NADH oxidase activity characteristic of the cytoplasmic membrane, were localized in the cytoplasmic membrane fraction.  Ref: 9159515 Mol Microbiol, 1997
- When ExbB and  **ExbD** , as well as their cross talk competent homologues ToIQ and ToIR, were absent,  **TonB**  was found associated with the outer membrane.  Ref: 9159515 Mol Microbiol, 1997
- **TonB** , ExbB and  **ExbD**  are only required for outer membrane transport.  Ref: 7688295 EMBO J, 1993
- Energy coupled transport and signal transduction through the gram negative outer membrane via  **TonB**  ExbB  **ExbD**  dependent receptor proteins.  Ref: 7654405 FEMS Microbiol Rev, 1995
- TolQ TolR interacted through  **TonB** , and ExbB  **ExbD**  interacted through TolA with the outer membrane receptors and colicins.  Ref: 8316079 Mol Microbiol, 1993
